# The Role of F-Box Proteins during Viral Infection

**DOI:** 10.3390/ijms14024030

**Published:** 2013-02-18

**Authors:** Régis Lopes Correa, Fernanda Prieto Bruckner, Renan de Souza Cascardo, Poliane Alfenas-Zerbini

**Affiliations:** 1 Department of Genetics, Federal University of Rio de Janeiro, Rio de Janeiro, RJ 21944-970, Brazil; E-Mails: regis@biologia.ufrj.br (R.L.C.); renan_cascardo@yahoo.com.br (R.S.C.); 2 Department of Microbiology/BIOAGRO, Federal University of Viçosa, Viçosa, MG 36570-000, Brazil; E-Mail: fepbruckner@yahoo.com.br

**Keywords:** F-box, SCF complex, virus-host interactions, viral infection

## Abstract

The F-box domain is a protein structural motif of about 50 amino acids that mediates protein–protein interactions. The F-box protein is one of the four components of the SCF (SKp1, Cullin, F-box protein) complex, which mediates ubiquitination of proteins targeted for degradation by the proteasome, playing an essential role in many cellular processes. Several discoveries have been made on the use of the ubiquitin–proteasome system by viruses of several families to complete their infection cycle. On the other hand, F-box proteins can be used in the defense response by the host. This review describes the role of F-box proteins and the use of the ubiquitin–proteasome system in virus–host interactions.

## 1. Introduction

F-box proteins (FBPs) are a large and diverse family of proteins present in all eukaryotes that are characterized by presence of the F-box domain [1]. There are 68 predicted genes coding FBPs in humans, 11 in yeast, 326 in *Caenorhabditis elegans*, about 700 in *Arabidopsis thaliana* and 600 in rice (*Oriza sativa*) [2–6]. Most of the characterized FBPs are components of the SCF (SKp1, Cullin, F-box protein) E3 ubiquitin–ligase complex. The SCF E3 ubiquitin–ligase complex participates in the recognition and recruitment of target proteins for ubiquitination and degradation by the ubiquitin 26S proteasome system (UPS). However, FBPs acting independently of the SCF complex have also been identified [7–9].

FBPs are structurally and functionally diverse, and their activity is crucial for selecting proteins that will be targeted by the SCF complex. The F-box domain consists of a conserved sequence of about 50 amino acids implicated in the interaction with core members of the complex [1, 10, 11]. Besides the F-box domain, other domains and motifs related to protein–protein interactions are usually present in the *C*-terminal region of FBPs, such as leucine rich repeats (LRR), WD40 repeats (WD), Kelch repeats, proline-rich and others. In general, FBPs are subdivided and classified according to these additional motifs. In mammal and yeast, the LLR and WD domains are frequently found. In humans, for example, the FBPs FBXL and FBXW have LLR and WD domains, respectively, while FBXO lacks both of them [12]. FBPs from plants have additional domains, but their identity and distribution are consistently different from their mammalian counterparts. In *Arabidopsis*, Kelch repeats are the most common domain found in FBPs. However, most of the predicted FBPs in *Arabidopsis* lack any other extra domain [6]. In rice, genome analysis revealed that FBPs have a wide plethora of additional domains, some of them with unknown function [3]. Due to the diversification of additional domains, the FBPs in the model plants *A. thaliana* and rice are divided into 19 and 10 groups, respectively [3, 6].

## 2. SFC Complex and the UPS

The UPS has an important role in protein turnover in the cell and in the regulation of different processes, such as cell division, development, hormone signaling, self-incompatibility and circadian cycle [13– 17]. The system degrades specific proteins that are post-translationally tagged by polyubiquitination. The binding of ubiquitins in a sequential and specific manner occurs in a cascade of events that involves the action of three enzymes or protein complexes, E1 (ubiquitin-activating enzyme), E2 (ubiquitin-conjugating enzyme) and E3 (ubiquitin ligase). Ubiquitin monomers are initially activated by binding to E1. Ubiquitin is then transferred to E2, and is finally transferred to the target protein by the action of E3. This process is repeated until a polyubiquitin tail signal is formed, driving the protein to degradation. Monoubiquitination is implicated in other cellular processes, such as relocalization of proteins [18].

E3 is the most diverse component of the UPS system, and is responsible for correctly recognizing the substrate target protein. There are different types of E3 complexes and the SCF is the most common and best understood. The SCF complex is composed of four proteins, S-phase kinase-associated protein-1 (SKP1), a CULLIN, a RING-finger protein (RBX1/ROC1/HRT1) and a FBP. The CULLIN protein is the backbone of the complex and binds to the RING-finger and SKP1 proteins on its *C*-terminal and *N*-terminal regions, respectively, forming a C-shaped structure [19, 20]. The RING-finger protein interacts with the ubiquitin-conjugated E2 enzyme and the FBP binds to SKP1 across the F-box domain. In order to be recognized by the FBP, substrate proteins are usually phosphorylated at specific amino acids. Additional FBP domains interact with the substrate protein and contribute to the correct positioning of the target in the active center of complex, where it is ubiquinated [21].

The swap of FBPs within the same SCF complex allows the recognition of a broad range of substrates, providing a great versatility of functions to the complex. Many FBPs are transcriptionally regulated for expression to be temporally and tissue specific, or in response to (a)biotic stressors [3] Furthermore, FBPs themselves can be degraded by the UPS system and some non-FBP substrates are known to compete with FBPs for the SCF’s active site, avoiding the self-ubiquitination of F-box proteins by the SFC complex [22]. Some FBPs can also be regulated by the interaction with small molecules to be able to bind to the SCF complex [16].

The diverse functions of FBPs act through the SCF complex, by binding to SKP1 or SKP1-like proteins, or in association with other complexes [7]. Two different classes of E3 ligases are involved in this process, the APC/C (anaphase promoting complex/cyclosome) and the SCF complexes [23]. APC/C is the major regulator, inducing proteolysis of inhibitors of the next phase of the cycle before the checkpoints. FBPs have an important role in cell cycle regulation by promoting the degradation of regulators of cell cycle progression, such as cyclins and cyclin-dependent kinases (CDKs). In mammals, where the process is better understood, many FBPs, such as SKP2, FBXW7 and β-TrCP have a crucial function in controlling the passage of one mitotic phase to the next [24, 25]. The S-phase kinase-associated protein 2 (SKP2) forms an SCF complex with SKP1, CULLIN-1 and RBX1 that is known to target more than twenty proteins for ubiquitin-mediated degradation. With this vast repertoire of substrates, SCF^SKP2^ is critical in controlling cell cycle progression, G1/S transition, apoptosis, avoidance of DNA re-replication, V(D)J recombination, cell proliferation potential, among other processes [26]. The SKP2 substrate P27 is an inhibitor of cell cycle progression and is considered a key piece in the regulation of the G1-S transition [27–29]. FBXW7 has an opposite effect by degrading activators of cell cycle progression, including c-MYC [30], NOTCH [31], c-JUN [32] and CYCLIN E [33]. β-TrCP mediates the ubiquitination of EMI1, an APC/C complex inhibitor, controlling the transition from phase G2 to M [34] and is also involved in the degradation of a repressor of the type 1 interferon immune response [35].

In plants, FBPs are also known to be implicated in the regulation of the cell cycle, hormone signaling and development. TIR1 (transport inhibitor response1) was one of first FBPs characterized in plants [36]. It is a positive regulator of the auxin hormone response, and complete or partial mutations in the *Tir1* gene lead to an auxin-resistance phenotype [37]. Recently, it was demonstrated that auxin and inositol hexakisphosphate molecules bind directly to TIR1 and contribute to its binding to target proteins [38]. This is an interesting demonstration of how FBPs can be regulated by small molecules. The control of other phytohormones is made in a similar manner. For example, jasmonate signaling is regulated by a TIR1-related FBP called COI1 (Coronatine Intensitive1) [39, 40], and the process is also dependent on a small molecule co-factor [41].

## 3. The UPS in Viral–Host Interactions

To successfully replicate in infected hosts, viruses need to create a suitable cell environment to complete their life cycle. Since the UPS plays a critical role in many cellular processes, it is not surprising that several unrelated viruses have evolved convergent strategies to exploit this mechanism. In recent years, a number of proteins encoded by both animal and plant viruses have been reported to redirect host ubiquitination components to their own needs. As expected, most ubiquitin-interfering viral proteins act on cellular pathways that rely on the SCF system for their regulation, such as innate immunity, signal transduction and the cell cycle pathways (Table 1).

### 3.1. Disruption of the Innate Immunity and Cell Signaling by Viruses

Several unrelated animal viruses encode proteins that interfere with signal transduction pathways involved in induction or amplification of the immune response, particularly the innate response driven by type I interferon (IFNα/β) [75]. The rotavirus protein NS1 is an example of how viruses can manipulate the interferon expression through the manipulation of the SCF system. The transcription factor nuclear factor kappa B (NFκB) is required for the induction of IFNβ [76], but its subunits are held inactive in the cytoplasm when associated with inhibitors of κB (IκB). Phosphorylation of IκB by IκB kinases (IKKα/β) results in its rapid ubiquitination by the E3 ligase Skp1/Cul1/F-box complex and subsequent degradation by the 26S proteasome, promoting the transcription of NFκB target genes, including IFNβ [77]. The substrate IκB is normally recognized by the FBP β-transducin repeat containing protein (β-TrCP) present in the SCF complex (SCF^β−TrCP^) [35]. During rotavirus infection, however, β-TrCP is degraded in a proteasome-dependent manner, stabilizing the expression of the phosphorylated IκBα and therefore maintaining NFκB in its inhibited state. The expression of the rotavirus protein NSP1 is sufficient to induce this effect [42].

The strategy of interferon inhibition is also adopted by other viruses. The *Human immunodeficiency virus type-1* (HIV-1) Vpu protein binds to βTrCP and CD4, the major cellular receptor for HIV-1 [78], resulting in the degradation of CD4. Competition with IκBα for βTrCP binding is believed to stabilize the former protein, promoting, NFκB inhibition [43]. It has been recently shown that the open reading frame 2 (ORF2) protein from the human *Hepatitis E virus* [44] and the large T antigen (TAg) tumor protein of JC virus (JCV) [45] may control IκBα levels in a similar fashion. The V proteins from the paramyxoviruses *Simian virus 5* (SV5) and type II *Human parainfluenza virus* (HPIV2) are able to disrupt the type I IFN signaling by inhibiting the latent signal transducer and activator of transcription (STAT) proteins, STAT1 and STAT2 [46]. The STAT proteins are able to dimerize and, together with IRF9, form a transcriptional factor that activates the expression of interferon-responsive antiviral genes [79]. SV5 and HPIV2 V proteins mediate the ubiquitin-dependent degradation of STAT proteins in a complex with the cullin protein CUL4 and the damaged DNA binding protein DDB1 [46]. The complex therefore acts as an E3 ligase, leading to the degradation of key proteins in the type 1 IFN signaling.

Interference with NFκB activity is also a strategy adopted by some members of the family *Poxviridae*, the masters in the de-regulation of the host’s ubiquitin–proteasome system. Poxviruses have several ankyrin proteins containing regions resembling the F-box domain [80]. Some of the so-called PRANC (pox protein repeat of ankyrin *C*-terminus)/F-box proteins have been shown to interact with cullin proteins and possibly mediate substrate-specific degradation [49]. The *Myxoma virus* M150 [47] and the *Cowpox virus* CP77 [48] proteins, for example, interact with the NFκB subunit p65, inhibiting the transcription of inflammatory cytokines. The G1R protein from *Variola virus* and its orthologs in *Cowpox virus*, *Monkeypox virus*, and *Ectromelia virus* are able to target another subunit (p105) of NFκB [81]. Poxviruses have a second class of proteins with BTB/Kelch domains that also function as substrate-specific adaptors to target proteins for ubiquitination [82–84, 50]. The BTB domain of those proteins is able to bind to CUL3 cullin proteins, and the Kelch domain acts by recruiting the substrate-target protein. The function of those proteins, however, is still unclear. They could act merely by sequestering host CUL3 from their normal complexes or rather form an active E3 ligase complex to deliberately manipulate the cellular protein machinery.

Apart from having F-box-like and BTB/Kelch proteins, poxviruses developed three other strategies for interfering with the host’s UPS: expression of ubiquitin homologs, ubiquitin ligases and APC/C-like proteins [49]. Although the poxvirus ubiquitin homologs identified so far are very similar to human ubiquitins, their functions in the infection process is still unclear [85– 87]. The two types of ubiquitin ligases encoded by poxviruses, RING-CH (MARCH) and the really interesting new gene (RING) finger protein, however, seem to function by degrading surface proteins involved in the immune response, such as MHC class I, MHC class II, CD4, CD95 and ALCAM [51, 88– 91]. Immune modulation by viral-encoded E3 ligases is also observed in *Kaposi’s sarcoma-associated herpesvirus* (KSHV). Proteins K3 and K5 from KSHV are able to target several surface proteins for degradation, including MHC class I, B7-2, ICAM-1, CD1d, PECAM-1, ALCAM, IFN-gR1, MICA/B, AICL and vascular endothelial cadherin (VE-cadherin) [92–99].

Plant viruses are also able to subvert cell signaling pathways. Geminiviruses are sensitive to the jasmonate hormone and, recently, it was found that the viral protein C2 is able to interfere with this hormone’s signaling by binding to CSN5, the catalytic subunit of the COP9 signalosome (CSN) complex [67]. CSN is a complex of proteins implicated on the removal of ubiquitin-like proteins called RUB (related to ubiquitin) from the CUL1 subunit of SCF complexes [100, 101]. The derubylation of CUL1 is essential for the SCF’s proper function. Since CUL1-related E3 ligases are involved in the regulation of many phytohormones, including jasmonate, the interference with that process is a powerful strategy for promoting viral infection. Indeed, transcriptomic analysis reveals a clear suppression of jasmonate responses in C2-expressing plants, and jasmonate treatment reduces the susceptibility to geminivirus infection [102].

Plants’ innate immunity, which usually leads to the hypersensitive response (HR), is also targeted by plant viruses through the deregulation of the UPS. HR is a type of innate defense system used by plants for avoiding the spread of pathogens. The mechanism is characterized by the rapid induction of cell death in infected and nearby cells, preventing the progression of the infection process [103]. The P25 protein from the benyvirus *Beet necrotic yellow vein virus* (BNYVV) is able to bind to a host Kelch-type FBP [68]. The interaction seems to be important for resistance suppression, since the transient expression of the Kelch FBP alone in *Nicotiana bethamiana* leaves was enough for inducing HR [69].

### 3.2. Disruption of the Cellular Cycle by Viruses

The disturbance of cell cycle proteins through the ubiquitin–proteasome system is another common theme for promoting viral replication. In animals, as well known mechanism by which poxviruses manipulate the host’s SCF machinery is by encoding components of the ubiquitin ligase APC/C. The PACR (poxvirus APC/cyclosome regulator) protein from the poxvirus is able to mimic the RING-H2 subunit APC11 of the APC/C [104]. However, unlike APC11, PACR has no ligase activity, inhibiting the complex. By preventing APC/C function, PACR may prompt cells to enter the S-phase of the cell cycle, where viral replication is stimulated by host factors.

Apart from poxviruses, this mechanism is frequently observed in several cancer-associated viruses and the P53 tumor suppressor protein is a common target for several viral proteins. P53 expression is associated with cell cycle arrest, DNA repair and apoptosis [105]. The nuclear antigen 3C (EBNA3C) protein from the herpesvirus *Epstein-barr virus* (EBV) is able to interfere with P53 function by indirectly targeting the MDM2 protein [52]. MDM2 is a negative regulator of P53 [106]. The protein has a P53-specific ubiquitin ligase domain and is able to target the tumor suppressor protein to degradation. MDM2 itself is also regulated by ubiquitination [107, 108]. By deubiquitinating MDM2, EBNA3C is able to stabilize the protein. Furthermore, EBNA3C enhances MDM2 ubiquitin ligase activity towards P53, providing robustness to the inhibition [52]. Two other proteins from *Adenovirus type 5* (Ad5) and one protein from *Human papillomavirus* (HPV) are also able to downregulate P53 activity. Ad5’s proteins E1B-55K and E4-ORF6, together with other proteins, form a ubiquitin–ligase complex that directs P53 and other targets to degradation [55–57]. Substrate recognition is believed to be mediated by E1B-55K, whereas E4-ORF6 is involved in the assembly of the complex. Likewise, the oncoprotein E6 from HPV forms a complex with other proteins that is able to ubiquitinate and degrade P53 [59]. By disrupting the P53-mediated G1/S-transition checkpoint, all these viral proteins, together with other factors, allow entry into the S-phase, potentially leading to the formation of tumors.

The FBP SKIP2 is also frequently involved in viral–host circuits. Besides having a role in the regulation of P53, the EBV protein EBNA3C mentioned above is also able to recruit the SCF^SKP2^ complex to promote the degradation of two of its targets: the tumor suppressor P27 and Retinoblastoma (RB) proteins [53, 54]. EBNA3C recruits SCF^SKP2^ to CYCLIN A/CDK2 complexes and serves as a scaffold that facilitates the ubiquitination of the kinase inhibitor P27 [53]. Downregulation of P27 ultimately leads to an increase in kinase activity, stimulating cell division. EBNA3C-directed enhancement of SCF^SKP2^ ubiquitination of RB proteins is also observed in EBV-transformed cells [54]. RB is a protein involved in the repression of proto-oncoproteins of the E2F family [109]. Interestingly, EBNA3C has some degree of sequence similarity with the HPV-16 oncoprotein E7 and the E1A protein from *Simian virus 40*, two proteins that are also known to target RB proteins for 26S proteasome degradation [110]. Therefore, HPV oncoproteins E6 and E7, by targeting two proteins involved in cell cycle arrest, P53 and RB, respectively, cooperate to stimulate entry into the S-phase. Curiously, E7 itself can be targeted for degradation by the SCF^SKP2^ complex [60]. An extra layer of regulation in this circuit of HPV’s oncoproteins is mediated by the E2 viral protein. E2 directly binds to the promoter sequence of E6 and E7 and negatively regulates their expression [111, 112]. E2 proteins exhibit short half-life times due to degradation by the ubiquitin/proteasome pathway [113–116]. Degradation of E2 occurs via SCF^SKP2^, since silencing of SKP2 induces stabilization of E2 [113].

There are additional examples of viral proteins interfering with SCF^SKP2^ activity. The HBV X protein (HBx) of *Hepatitis B virus* (HBV) is able to increase the stability of the proto-oncoprotein c-MYC by inhibiting its SKP2-mediated degradation [61]. HBX also promotes the inhibition of SCF-induced ubiquitination of the pituitary tumor-transforming gene 1 (PTTG1)-encoded protein, a human securin protein implicated in inhibition of sister chromatid separation during mitosis. The interaction between PTTG1 and the SCF ubiquitin–ligase complex occurs through a mechanism involving protein–protein interactions of HBx with PTTG1 and/or SCF [62]. Recently, it was shown that the viral interferon-regulatory factor-3 (vIRF-3) protein from the herpesvirus KSHV can associate with SCF^SKP2^ and also promote the expression of the proto-oncogene c-*Myc*, encouraging cell growth [63]. vIRF-3 activity is based on the fact that SCF^SKP2^ not only ubiquitinates and degrades c-MYC, but its activity is also important for the activation of the transcription of c-MYC targets. vIRF-3 is able to bind to SKP2 and enhances the transcription of c-MYC-regulated genes [63]. The viral protein is also able to interfere with P53 [64] and NFκB [65] activities during the infection process.

A second E3 ligase associated in the regulation of the cell cycle, SCF^FBW7^, is also targeted by viral proteins. FBW7, also known as SEL-10, hCDC4 and hAGO, is the F-box component of the SCF^FBW7^, a complex involved in the ubiquitin-dependent regulation of several proto-oncoproteins. The latency associated nuclear antigen (LANA) from the herpesvirus KSHV can compete with NOTCH for FBW7 binding, therefore, avoiding its degradation [66]. Cell growth stimulation through SCF^FBW7^ inhibition is also achieved by the adenoviral protein E1A. The direct interaction of EA1 with components of the SCF^FBW7^ does not disturb its assembly; however, its ubiquitin-ligation activity is compromised by a mechanism not completely understood [58].

RB proteins are also targeted by proteins encoded by plant viruses. The 20 kDa CLINK protein from members of the plant viral family *Nanoviridae* binds to RB proteins through its LxCxE motif. CLINK is an FBP that interacts with SKP1 and enhances viral replication during nanovirus infection and has the ability to alter the activity of pRB. The inactivation of pRb by CLINK induces cells with the cell cycle progression inactive to enter in endoreduplication cycles creating a cellular environment favorable for viral replication [70]. It is suggested that CLINK leads to the degradation of RB via its F-box domain, releasing the transcription factor E2F family to activate cell cycle progression-related genes.

### 3.3. Suppression of RNA Silencing by Viral F-Box-Like Proteins

The interaction between pathogens and the host’s SCF machinery has also been observed in members of the plant viral family *Luteoviridae*. Viruses belonging to the family are divided into three genera: *Luteovirus*, *Polerovirus* and *Enamovirus*. The P0 proteins from several members of the genus *Polerovirus* and the sole member of the genus *Enamovirus* has been shown to be suppressors of the plant’s RNA silencing defense mechanism [71, 117, 118]. Although the protein lacks typical domains such as C-terminal leucine-rich repeats or WD-40 repeats, P0 has a central region resembling the F-box domain and is thus considered an F-box-like protein [71, 119].

RNA silencing is a conserved mechanism that regulates gene expression in a sequence-specific manner. During the process, double-strand RNA (dsRNA) molecules are recognized and degraded by members of the Dicer family, a type III RNase [120]. The cleavage gives rise to double-stranded small RNAs (sRNAs) ranging from 21 to 26 nucleotides that are loaded into RNA-induced silencing complexes (RISC) [121, 122]. RISC can drive gene regulation at the post-transcriptional level by targeting mRNAs for degradation or inhibiting protein translation, or at the transcriptional level by inducing DNA methylation [123]. The specificity of the regulation is given by the sRNA bound to RISC and Argonaute proteins are the “slicer” component of the complex [124].

In the model plant *A. thaliana*, there are four Dicer-like (DCL1 to DCL4) and 10 Argonaute (AGO1 to AGO10) proteins. This vast repertoire of silencing-related proteins allowed the diversification and specialization of the sRNA-directed pathways. Viral dsRNA structures can be degraded by DCL4, DCL2 and DCL3 in *Arabidopsis*, giving rise to primary sRNAs from 21 to 24 nucleotides [71, 125–128]. Virus-derived sRNAs can both drive viral RNA degradation through AGO1/2/4/5/7-containing complexes or recruit RDR proteins for *de novo* dsRNA synthesis [128–130], generating secondary sRNAs [131]. Viruses, as a counter defense, evolved diverse and unrelated strategies to block the plant’s RNA degradation machinery [132].

The silencing suppressor P0, absent in the genus *Luteovirus*, is encoded by the first ORF in the two other genera of the family. Partially due to its lack of similarity with any other known viral protein, its function remained a mystery for years. Transgenic potato plants overexpressing the P0 from *Potato leafroll virus* (PLRV), the type member of the genus *Polerovirus*, produced the first clues that the protein could somehow modulate symptom expression [133]. However, it was not until the last decade that additional experiments uncovered its silencing suppression activity for the first time [118]. The presence of an intact F-box-like-domain proved to be essential for its activity [71, 119]. Accordingly, direct interactions between the Polerovirus P0 and the Arabidopsis SKP2 protein or its yeast homolog SKP1 were detected by yeast two-hybrid experiments [119].

Agro-infiltration experiments done in the 16c GFP-expressing line of *Nicotiana benthamiana* revealed that P0 is able to suppress both local and systemic silencing, although at variable degrees among different viruses and isolates [71, 117–119, 134, 135]. And similar to *ago1* mutants, the expression of P0 in either *N. benthamiana* or Arabidopsis leads to a reduction in the levels of secondary siRNAs and a downregulation of several regulatory DCL1/AGO1-dependent microRNAs [71, 136–138]. In accordance, P0s can lead to AGO1 destabilization on both transient and stable expression experiments [71, 135, 136, 138]. More specifically, it was observed that the conserved PAZ and the adjacent ND domains were necessary for P0-mediated destabilization in all AGO proteins tested [136]. Although the PAZ domain is conserved in animal Argonaute homologs, the strong P0 from *Curcubit aphid-borne yellows virus* (CAbYV) is not able to suppress RNA silencing in adult *Drosophila* triggered by dsRNAs or sRNAs [139].

The exact mode of action of P0 is still under debate. Transient expression experiments indicate that the BWYV P0 is not able to target pre-loaded AGO1 complexes [137]. Although the protein seems to enhance the overall ubiquitination pattern of host proteins, its activity is not compromised in the presence of proteasome inhibitors [136, 138]. Since the Arabidopsis AGO1 itself is regulated by a host F-box protein (FBW2) [72], perhaps the suppressor might somehow interfere with AGO1’s natural ubiquitination process. Alternatively, the P0s might target other AGOs or interfere with other components of RISC which would ultimately lead to AGO1 destabilization. The latter hypothesis is supported by the fact that the expressions of P0s from the *Sobemovirus Potato leaf roll virus* and from *Enamovirus Pea enation mosaic virus-1* induce unrelated phenotypes in transgenic *Arabidopsis* plants when compared side by side [71]. The displayed phenotypes are fundamentally different from those observed in *ago1* mutant, indicating that the set of proteins targeted by P0 might be pathogen-specific.

AGO1 is also targeted for degradation in a proteasome-dependent manner by the movement protein P25 from the potexvirus *Potato virus X* (PVX) [73]. P25 binds not only to AGO1 but also to AGO2, AGO3 and AGO4. Although the P25-mediated destabilization of AGO can be blocked by treatment with proteasome inhibitors, it is still not known whether the protein can interact directly with components of the UPS, although the fact that PVX infection leads to an increase in SKP1 levels indirectly supports this idea [74, 140].

## 4. Conclusions

It is currently quite clear that the subversion of the host’s UPS is a common strategy for promoting viral infection. The diversity of mechanisms by which viruses can achieve this goal is enormous. There are viral proteins resembling or disturbing almost all known steps of the UPS. Actually, many aspects of the UPS itself were discovered through studies of viral pathogenicity factors. Here, we reviewed the action of viral proteins on innate immunity, cell signaling and in the control of the cell cycle in both animals and plants. The disturbance of the plant’s RNA silencing machinery by viruses was also discussed. Most viral proteins discovered so far deliberately manipulate the function of hub proteins involved in the control those mechanisms. By reducing defense mechanisms or promoting cell proliferation, viruses can reprogram the cell’s metabolism towards their own needs. Future research should focus on the discovery of new UPS components encoded by viruses, especially in plant viruses, where this topic remains poorly explored. The discovery of the targets of viral-encoded FBPs is crucial for providing new insights into this fascinating host–virus interaction.

## Figures and Tables

**Table 1:** Ubiquitin-interfering viral proteins and the use of the ubiquitin–proteasome system by viruses of several families to complete their infection cycle.

Virus	Viral protein	Mechanism of action	References
**Animal viruses**			
*Rotavirus*	NSP1	NSP1 prevents degradation of IκBα and maintains NFκB in its inhibited state by induction of β-TrCP degradation by UPS system	[42]
*Human immunodeficiency virus type-1* (HIV-1)	Vpu	Vpu interacts with CD4 and β-TrCP triggering CD4 degradation by UPS system	[43]
*Hepatitis E virus* (HEV)	ORF2	ORF2 interacts with β-TrCP damaging SCF β-TrCP assembly. As a result, degradation of IκBα is inhibited and NFκB remains in its inhibited state	[44]
*JC virus*(JCV)	TAg	TAg interacts with β-TrCP and this interaction effect is unknown	[45]
*Simian virus 5* (SV5) and type II *Human parainfluenza virus* (HPIV2)	V proteins	V proteins interact with CUL4 and DDB1 and this complex acts as an E3 ligase, leading to the degradation by UPS system of STAT proteins and key proteins that act in the type 1 IFN signaling	[46]
*Myxoma virus*	M150	Interacts with cullin and with p65 subunit of NFκB, triggering its degradation	[47]
*Cowpox virus*	CP77	Interacts with cullin and with p65 subunit of NFκB, triggering its degradation	[48]
Variola virus: *Cowpox virus*, *Monkeypox virus*, and *Ectromelia virus*	G1R	Interacts with cullin and with p105 subunit of NFκB, triggering its degradation	[49]
*Poxvirus*	proteins with BTB/Kelch domains	Interacts with CUL3 by BTB domain and with target protein by Kelch domain. The functions of these target proteins, however, is still unclear	[50]
*Poxvirus*	poxvirus APC/cyclosome regulator (PACR)	Interacts with APC/C proteins replacing the RING-H2 subunit APC11. PACR has no ligase activity, inhibiting the complex activity	[51]
*Epstein-barr virus* (EBV)	EBNA3C	Interferes in ubiquitination of p53 and MDM2, leading to degradation and stabilization of these proteins, respectively, inducing tumors; recruits SCF^SKP2^ complex to promote the degradation of the tumor suppressor P27 and Retinoblastoma (RB) proteins	[52–54]
*Adenovirus type 5* (Ad5)	E1B-55K and E4-ORF6	Participate, with other proteins, of ubiquitin–ligase complex that directs P53 and other targets to degradation	[55–57]
*Adenovirus type 5* (Ad5)	E1A	Interacts directly with components of the SCF^FBW7^ and undertakes its ubiquitin ligation activity by a mechanism not completely understood	[58]
*Human papillomavirus* (HPV)	E6 and E7	Interact with other proteins forming a complex that is able to ubiquitinate and degrade P53 and Retinoblastoma proteins, respectively	[59, 60]
*Hepatitis B virus* (HBV)	HBX	Increases the stability of c-MYC by inhibiting its SKP2-mediated degradation; Inhibits SCF-induced ubiquitination of the pituitary tumor-transforming gene 1 (PTTG1)-encoded protein by interaction with PTTG1 and the SCF ubiquitin–ligase complex	[61, 62]
herpesvirus KSHV	viral interferon-regulatory factor-3 (vIRF-3) protein	vIRF-3 enhances the transcription of c-MYC-regulated genes by interaction with SKP2. Interferes with P53 and NFκB activities during the infection process	[63–65]
herpesvirus KSHV	latency associated nuclear antigen	can compete with NOTCH for FBW7 binding, therefore, avoiding its degradation	[66]
**Plant viruses**			
*Geminivirus*	C2	Interacts with CSN5, the catalytic subunit of the COP9 signalosome (CSN) complex, interfering in phytohormone regulation and suppressing responses mediated by jasmonate	[67]
*Beet necrotic yellow vein virus* (BNYVV)	P25	P25 binds to a host Kelch-type FBP and probably suppresses HR responses	[68, 69]
*Nanovirus*	CLINK	Acts as an FBP that is able to interact with RB proteins and SKP1 and enhances viral replication during nanovirus infection in a mechanism that probably inducing RB proteins degradation	[70]
*Polerovirus*	P0	It is able to interact with SKP2 and acts as silencing suppressor probably triggering AGO1 to degradation by UPS system. The action mechanism remains unclear	[71]
*Potato virus X* (PVX)	P25	Binds to AGO1, AGO2, AGO3 and AGO4 and triggers AGO1 to degradation in a proteasome	[72–74]

## References

[1] Bai C., Sen P., Hofmann K., Ma L., Goebl M., Harper J.W., Elledge S.J. (1996). SKP1 connects cell cycle regulators to the ubiquitin proteolysis machinery through a novel motif, the F-box. Cell.

[2] Gagne J.M., Downes B.P., Shiu S.H., Durski A.M., Vierstra R.D. (2002). The F-box subunit of the SCF E3 complex is encoded by a diverse superfamily of genes in Arabidopsis. Proc. Natl. Acad. Sci. USA.

[3] Jain M., Nijhawan A., Arora R., Agarwal P., Ray S., Sharma P., Kapoor S., Tyagi A.K., Khurana J.P. (2007). F-box proteins in rice. Genome-wide analysis, classification, temporal and spatial gene expression during panicle and seed development, and regulation by light and abiotic stress. Plant Physiol.

[4] Bai J.R., Jia X., Liu K.F., Wang D.W. (2004). Cloning and characterization of the coding sequences of the 1Ay high molecular weight glutenin subunit genes from Triticum urartu. Acta Botanica Sinica.

[5] Kipreos E.T., Pagano M (2000). The F-box protein family. Genome Biol..

[6] Kuroda H., Takahashi N., Shimada H., Seki M., Shinozaki K., Matsui M. (2002). Classification and expression analysis of Arabidopsis F-box-containing protein genes. Plant Cell Physiol.

[7] Hermand D. (2006). F-box proteins: more than baits for the SCF?. Cell Division.

[8] Hermand D., Bamps S., Tafforeau L., Vandenhaute J., Makela T.P. (2003). Skp1 and the F-box protein Pof6 are essential for cell separation in fission yeast. J. Biol. Chem.

[9] Clifford R., Lee M.H., Nayak S., Ohmachi M., Giorgini F., Schedl T. (2000). FOG-2, a novel F-box containing protein, associates with the GLD-1 RNA binding protein and directs male sex determination in the *C. elegans* hermaphrodite germline. Development.

[10] Feldman R.M., Correll C.C., Kaplan K.B., Deshaies R.J. (1997). A complex of Cdc4p, Skp1p, and Cdc53p/cullin catalyzes ubiquitination of the phosphorylated CDK inhibitor Sic1p. Cell.

[11] Skowyra D., Craig K.L., Tyers M., Elledge S.J., Harper J.W. (1997). F-box proteins are receptors that recruit phosphorylated substrates to the SCF ubiquitin-ligase complex. Cell.

[12] Jin J., Cardozo T., Lovering R.C., Elledge S.J., Pagano M., Harper J.W. (2004). Systematic analysis and nomenclature of mammalian F-box proteins. Genes Dev.

[13] Gusti A., Baumberger N., Nowack M., Pusch S., Eisler H., Potuschak T., De Veylder L., Schnittger A., Genschik P. (2009). The Arabidopsis thaliana F-box protein FBL17 is essential for progression through the second mitosis during pollen development. PLoS One.

[14] McClure B., Cruz-Garcia F., Romero C. (2011). Compatibility and incompatibility in S-RNase-based systems. Ann. Bot.

[15] Pauwels L., Goossens A. (2011). The JAZ proteins: A crucial interface in the jasmonate signaling cascade. Plant Cell.

[16] Somers D.E., Fujiwara S. (2009). Thinking outside the F-box: novel ligands for novel receptors. Trends Plant Sci.

[17] Souer E., Rebocho A.B., Bliek M., Kusters E., de Bruin R.A., Koes R. (2008). Patterning of inflorescences and flowers by the F-Box protein DOUBLE TOP and the LEAFY homolog ABERRANT LEAF AND FLOWER of petunia. Plant Cell.

[18] Kerscher O., Felberbaum R., Hochstrasser M. (2006). Modification of proteins by ubiquitin and ubiquitin-like proteins. Ann. Rev. Cell Dev. Biol.

[19] Orlicky S., Tang X., Willems A., Tyers M., Sicheri F. (2003). Structural basis for phosphodependent substrate selection and orientation by the SCFCdc4 ubiquitin ligase. Cell.

[20] Zheng N., Schulman B.A., Song L., Miller J.J., Jeffrey P.D., Wang P., Chu C., Koepp D.M., Elledge S.J., Pagano M. (2002). Structure of the Cul1-Rbx1-Skp1-F boxSkp2 SCF ubiquitin ligase complex. Nature.

[21] Schulman B.A., Carrano A.C., Jeffrey P.D., Bowen Z., Kinnucan E.R., Finnin M.S., Elledge S.J., Harper J.W., Pagano M., Pavletich N.P. (2000). Insights into SCF ubiquitin ligases from the structure of the Skp1-Skp2 complex. Nature.

[22] Zhou P., Howley P.M. (1998). Ubiquitination and degradation of the substrate recognition subunits of SCF ubiquitin-protein ligases. Mol. Cell.

[23] Skaar J.R., Pagano M. (2009). Control of cell growth by the SCF and APC/C ubiquitin ligases. Curr. Opin. Cell Biol.

[24] Frescas D., Pagano M. (2008). Deregulated proteolysis by the F-box proteins SKP2 and beta-TrCP: tipping the scales of cancer. Nat. Rev. Cancer.

[25] Welcker M., Clurman B.E. (2008). FBW7 ubiquitin ligase: a tumour suppressor at the crossroads of cell division, growth and differentiation. Nat. Rev. Cancer.

[26] Chan C.H., Lee S.W., Wang J., Lin H.K. (2010). Regulation of Skp2 expression and activity and its role in cancer progression. Sci. World J.

[27] Carrano A.C., Eytan E., Hershko A., Pagano M. (1999). SKP2 is required for ubiquitin-mediated degradation of the CDK inhibitor p27. Nat. Cell Biol.

[28] Sutterluty H., Chatelain E., Marti A., Wirbelauer C., Senften M., Muller U., Krek W. (1999). p45SKP2 promotes p27Kip1 degradation and induces S phase in quiescent cells. Nat. Cell Biol.

[29] Tsvetkov L.M., Yeh K.H., Lee S.J., Sun H., Zhang H. (1999). p27(Kip1) ubiquitination and degradation is regulated by the SCF(Skp2) complex through phosphorylated Thr187 in p27. Curr. Biol.

[30] Welcker M., Orian A., Jin J., Grim J.E., Harper J.W., Eisenman R.N., Clurman B.E. (2004). The Fbw7 tumor suppressor regulates glycogen synthase kinase 3 phosphorylation-dependent c-Myc protein degradation. Proc. Natl. Acad. Sci. USA.

[31] Wu G., Lyapina S., Das I., Li J., Gurney M., Pauley A., Chui I., Deshaies R.J., Kitajewski J. (2001). SEL-10 is an inhibitor of notch signaling that targets notch for ubiquitin-mediated protein degradation. Mol. Cell Biol.

[32] Nateri A.S., Riera-Sans L., Da Costa C., Behrens A. (2004). The ubiquitin ligase SCFFbw7 antagonizes apoptotic JNK signaling. Science.

[33] Koepp D.M., Schaefer L.K., Ye X., Keyomarsi K., Chu C., Harper J.W., Elledge S.J. (2001). Phosphorylation-dependent ubiquitination of cyclin E by the SCFFbw7 ubiquitin ligase. Science.

[34] Guardavaccaro D., Kudo Y., Boulaire J., Barchi M., Busino L., Donzelli M., Margottin-Goguet F., Jackson P.K., Yamasaki L., Pagano M. (2003). Control of meiotic and mitotic progression by the F box protein beta-Trcp1 *in vivo*. Dev Cell.

[35] Nakayama K., Hatakeyama S., Maruyama S., Kikuchi A., Onoe K., Good R.A., Nakayama K.I. (2003). Impaired degradation of inhibitory subunit of NF-kappa B (I kappa B) and beta-catenin as a result of targeted disruption of the beta-TrCP1 gene. Proc. Natl. Acad. Sci. USA.

[36] Gray W.M., Kepinski S., Rouse D., Leyser O., Estelle M. (2001). Auxin regulates SCFTIR1-dependent degradation of AUX/IAA proteins. Nature.

[37] Ruegger M., Dewey E., Gray W.M., Hobbie L., Turner J., Estelle M. (1998). The TIR1 protein of Arabidopsis functions in auxin response and is related to human SKP2 and yeast grr1p. Genes Dev.

[38] Tan X., Calderon-Villalobos L.I., Sharon M., Zheng C., Robinson C.V., Estelle M., Zheng N. (2007). Mechanism of auxin perception by the TIR1 ubiquitin ligase. Nature.

[39] Katsir L., Schilmiller A.L., Staswick P.E., He S.Y., Howe G.A. (2008). COI1 is a critical component of a receptor for jasmonate and the bacterial virulence factor coronatine. Proc. Natl. Acad. Sci. USA.

[40] Thines B., Katsir L., Melotto M., Niu Y., Mandaokar A., Liu G., Nomura K., He S.Y., Howe G.A., Browse J. (2007). JAZ repressor proteins are targets of the SCF(COI1) complex during jasmonate signalling. Nature.

[41] Sheard L.B., Tan X., Mao H., Withers J., Ben-Nissan G., Hinds T.R., Kobayashi Y., Hsu F.F., Sharon M., Browse J. (2010). Jasmonate perception by inositol-phosphate-potentiated COI1-JAZ co-receptor. Nature.

[42] Graff J.W., Ettayebi K., Hardy M.E. (2009). Rotavirus NSP1 inhibits NFkappaB activation by inducing proteasome-dependent degradation of beta-TrCP: A novel mechanism of IFN antagonism. PLoS Pathog.

[43] Margottin F., Bour S.P., Durand H., Selig L., Benichou S., Richard V., Thomas D., Strebel K., Benarous R. (1998). A novel human WD protein, h-beta TrCp, that interacts with HIV-1 Vpu connects CD4 to the ER degradation pathway through an F-box motif. Mol. Cell.

[44] Surjit M., Varshney B., Lal S.K. (2012). The ORF2 glycoprotein of hepatitis E virus inhibits cellular NF-kappaB activity by blocking ubiquitination mediated proteasomal degradation of IkappaBalpha in human hepatoma cells. BMC Biochem.

[45] Reviriego-Mendoza M.M., Frisque R.J. (2011). Interaction and co-localization of JC virus large T antigen and the F-box protein beta-transducin-repeat containing protein. Virology.

[46] Ulane C.M., Horvath C.M. (2002). Paramyxoviruses SV5 and HPIV2 assemble STAT protein ubiquitin ligase complexes from cellular components. Virology.

[47] Camus-Bouclainville C., Fiette L., Bouchiha S., Pignolet B., Counor D., Filipe C., Gelfi J., Messud-Petit F. (2004). A virulence factor of myxoma virus colocalizes with NF-kappaB in the nucleus and interferes with inflammation. J. Virol.

[48] Hsiao J.C., Chao C.C., Young M.J., Chang Y.T., Cho E.C., Chang W. (2006). A poxvirus host range protein, CP77, binds to a cellular protein, HMG20A, and regulates its dissociation from the vaccinia virus genome in CHO-K1 cells. J. Virol.

[49] Barry M., van Buuren N., Burles K., Mottet K., Wang Q., Teale A. (2010). Poxvirus exploitation of the ubiquitin-proteasome system. Viruses.

[50] Xu L., Wei Y., Reboul J., Vaglio P., Shin T.H., Vidal M., Elledge S.J., Harper J.W. (2003). BTB proteins are substrate-specific adaptors in an SCF-like modular ubiquitin ligase containing CUL-3. Nature.

[51] Guerin J.L., Gelfi J., Boullier S., Delverdier M., Bellanger F.A., Bertagnoli S., Drexler I., Sutter G., Messud-Petit F. (2002). Myxoma virus leukemia-associated protein is responsible for major histocompatibility complex class I and Fas-CD95 down-regulation and defines scrapins, a new group of surface cellular receptor abductor proteins. J. Virol.

[52] Saha A., Murakami M., Kumar P., Bajaj B., Sims K., Robertson E.S. (2009). Epstein-Barr virus nuclear antigen 3C augments Mdm2-mediated p53 ubiquitination and degradation by deubiquitinating Mdm2. J. Virol.

[53] Knight J.S., Sharma N., Robertson E.S. (2005). SCFSkp2 complex targeted by Epstein-Barr virus essential nuclear antigen. Mol. Cell Biol.

[54] Knight J.S., Sharma N., Robertson E.S. (2005). Epstein-Barr virus latent antigen 3C can mediate the degradation of the retinoblastoma protein through an SCF cellular ubiquitin ligase. Proc. Natl. Acad. Sci. USA.

[55] Querido E., Blanchette P., Yan Q., Kamura T., Morrison M., Boivin D., Kaelin W.G., Conaway R.C., Conaway J.W., Branton P.E. (2001). Degradation of p53 by adenovirus E4orf6 and E1B55K proteins occurs via a novel mechanism involving a Cullin-containing complex. Genes Dev.

[56] Querido E., Morrison M.R., Chu-Pham-Dang H., Thirlwell S.W., Boivin D., Branton P.E. (2001). Identification of three functions of the adenovirus e4orf6 protein that mediate p53 degradation by the E4orf6-E1B55K complex. J. Virol.

[57] Harada J.N., Shevchenko A., Pallas D.C., Berk A.J. (2002). Analysis of the adenovirus E1B-55K-anchored proteome reveals its link to ubiquitination machinery. J. Virol.

[58] Isobe T., Hattori T., Kitagawa K., Uchida C., Kotake Y., Kosugi I., Oda T., Kitagawa M. (2009). Adenovirus E1A inhibits SCF(Fbw7) ubiquitin ligase. J. Biol. Chem.

[59] Scheffner M., Huibregtse J.M., Vierstra R.D., Howley P.M. (1993). The HPV-16 E6 and E6-AP complex functions as a ubiquitin-protein ligase in the ubiquitination of p53. Cell.

[60] Oh K.J., Kalinina A., Wang J., Nakayama K., Nakayama K.I., Bagchi S. (2004). The papillomavirus E7 oncoprotein is ubiquitinated by UbcH7 and Cullin 1- and Skp2-containing E3 ligase. J. Virol.

[61] Kalra N., Kumar V. (2006). The X protein of hepatitis B virus binds to the F box protein Skp2 and inhibits the ubiquitination and proteasomal degradation of c-Myc. FEBS Lett.

[62] Molina-Jimenez F., Benedicto I., Murata M., Martin-Vilchez S., Seki T., Antonio Pintor-Toro J., Tortolero M., Moreno-Otero R., Okazaki K., Koike K. (2010). Expression of pituitary tumor-transforming gene 1 (PTTG1)/securin in hepatitis B virus (HBV)-associated liver diseases: evidence for an HBV X protein-mediated inhibition of PTTG1 ubiquitination and degradation. Hepatology.

[63] Baresova P., Pitha P.M., Lubyova B. (2012). Kaposi sarcoma-associated herpesvirus vIRF-3 protein binds to F-box of Skp2 protein and acts as a regulator of c-Myc protein function and stability. J. Biol. Chem.

[64] Rivas C., Thlick A.E., Parravicini C., Moore P.S., Chang Y. (2001). Kaposi’s sarcoma-associated herpesvirus LANA2 is a B-cell-specific latent viral protein that inhibits p53. J. Virol.

[65] Seo T., Park J., Lim C., Choe J. (2004). Inhibition of nuclear factor kappaB activity by viral interferon regulatory factor 3 of Kaposi’s sarcoma-associated herpesvirus. Oncogene.

[66] Lan K., Verma S.C., Murakami M., Bajaj B., Kaul R., Robertson E.S. (2007). Kaposi’s sarcoma herpesvirus-encoded latency-associated nuclear antigen stabilizes intracellular activated Notch by targeting the Sel10 protein. Proc. Natl. Acad. Sci. USA.

[67] Wei N., Serino G., Deng X.W. (2008). The COP9 signalosome: More than a protease. Trends Biochem. Sci.

[68] Thiel H., Varrelmann M. (2009). Identification of Beet necrotic yellow vein virus P25 pathogenicity factor-interacting sugar beet proteins that represent putative virus targets or components of plant resistance. Mol. Plant Microbe Interact.

[69] Thiel H., Hleibieh K., Gilmer D., Varrelmann M. (2012). The P25 Pathogenicity Factor of Beet necrotic yellow vein virus Targets the Sugar Beet 26S Proteasome Involved in the Induction of a Hypersensitive Resistance Response via Interaction with an F-box Protein. Mol. Plant Microbe Interact.

[70] Lageix S., Cattrice O., Deragon J.M., Gronenborn B., Pélissier T., Ramírez B.C. (2007). The nanovirus-encoded Clink protein affects plant cell cycle regulation through interaction with the retinoblastoma-related protein. J. Virol.

[71] Fusaro A.F., Correa R.L., Nakasugi K., Jackson C., Kawchuk L., Vaslin M.F., Waterhouse P.M. (2012). The Enamovirus P0 protein is a silencing suppressor which inhibits local and systemic RNA silencing through AGO1 degradation. Virology.

[72] Earley K., Smith M., Weber R., Gregory B., Poethig R. (2010). An endogenous F-box protein regulates ARGONAUTE1 in *Arabidopsis thaliana*. Silence.

[73] Chiu M.H., Chen I.H., Baulcombe D.C., Tsai C.H. (2010). The silencing suppressor P25 of Potato virus X interacts with Argonaute1 and mediates its degradation through the proteasome pathway. Mol. Plant Pathol.

[74] Ye C., Dickman M.B., Whitham S.A., Payton M., Verchot J. (2011). The unfolded protein response is triggered by a plant viral movement protein. Plant Physiol.

[75] Randall R.E., Goodbourn S. (2008). Interferons and viruses: an interplay between induction, signalling, antiviral responses and virus countermeasures. J. Gen. Virol.

[76] Sizemore N., Agarwal A., Das K., Lerner N., Sulak M., Rani S., Ransohoff R., Shultz D., Stark G.R. (2004). Inhibitor of kappaB kinase is required to activate a subset of interferon gamma-stimulated genes. Proc. Natl. Acad. Sci. USA.

[77] Kroll M., Margottin F., Kohl A., Renard P., Durand H., Concordet J.P., Bachelerie F., Arenzana-Seisdedos F., Benarous R. (1999). Inducible degradation of IkappaBalpha by the proteasome requires interaction with the F-box protein h-betaTrCP. J. Biol. Chem.

[78] Willey R.L., Maldarelli F., Martin M.A., Strebel K. (1992). Human immunodeficiency virus type 1 Vpu protein induces rapid degradation of CD4. J. Virol.

[79] Aaronson D.S., Horvath C.M. (2002). A road map for those who don’t know JAK-STAT. Science.

[80] Mercer A.A., Fleming S.B., Ueda N. (2005). F-box-like domains are present in most poxvirus ankyrin repeat proteins. Virus Genes.

[81] Mohamed M.R., Rahman M.M., Lanchbury J.S., Shattuck D., Neff C., Dufford M., van Buuren N., Fagan K., Barry M., Smith S. (2009). Proteomic screening of variola virus reveals a unique NF-kappaB inhibitor that is highly conserved among pathogenic orthopoxviruses. Proc. Natl. Acad. Sci. USA.

[82] Furukawa M., He Y.J., Borchers C., Xiong Y. (2003). Targeting of protein ubiquitination by BTB-Cullin 3-Roc1 ubiquitin ligases. Nat. Cell Biol.

[83] Geyer R., Wee S., Anderson S., Yates J., Wolf D.A. (2003). BTB/POZ domain proteins are putative substrate adaptors for cullin 3 ubiquitin ligases. Mol. Cell.

[84] Pintard L., Willis J.H., Willems A., Johnson J.L., Srayko M., Kurz T., Glaser S., Mains P.E., Tyers M., Bowerman B. (2003). The BTB protein MEL-26 is a substrate-specific adaptor of the CUL-3 ubiquitin-ligase. Nature.

[85] Afonso C.L., Tulman E.R., Lu Z., Oma E., Kutish G.F., Rock D.L. (1999). The genome of Melanoplus sanguinipes entomopoxvirus. J. Virol.

[86] Bawden A.L., Glassberg K.J., Diggans J., Shaw R., Farmerie W., Moyer R.W. (2000). Complete genomic sequence of the Amsacta moorei entomopoxvirus: Analysis and comparison with other poxviruses. Virology.

[87] Tulman E.R., Afonso C.L., Lu Z., Zsak L., Kutish G.F., Rock D.L. (2004). The genome of canarypox virus. J. Virol.

[88] Huang J., Huang Q., Zhou X., Shen M.M., Yen A., Yu S.X., Dong G., Qu K., Huang P., Anderson E.M. (2004). The poxvirus p28 virulence factor is an E3 ubiquitin ligase. J. Biol. Chem.

[89] Mansouri M., Bartee E., Gouveia K., Hovey Nerenberg B.T., Barrett J., Thomas L., Thomas G., McFadden G., Fruh K. (2003). The PHD/LAP-domain protein M153R of myxomavirus is a ubiquitin ligase that induces the rapid internalization and lysosomal destruction of CD4. J. Virol.

[90] Bartee E., Mansouri M., Hovey Nerenberg B.T., Gouveia K., Fruh K. (2004). Downregulation of major histocompatibility complex class I by human ubiquitin ligases related to viral immune evasion proteins. J. Virol.

[91] Senkevich T.G., Wolffe E.J., Buller R.M. (1995). Ectromelia virus RING finger protein is localized in virus factories and is required for virus replication in macrophages. J. Virol.

[92] Coscoy L., Ganem D. (2000). Kaposi’s sarcoma-associated herpesvirus encodes two proteins that block cell surface display of MHC class I chains by enhancing their endocytosis. Proc. Natl. Acad. Sci. USA.

[93] Ishido S., Choi J.K., Lee B.S., Wang C., DeMaria M., Johnson R.P., Cohen G.B., Jung J.U. (2000). Inhibition of natural killer cell-mediated cytotoxicity by Kaposi’s sarcoma-associated herpesvirus K5 protein. Immunity.

[94] Coscoy L., Ganem D. (2001). A viral protein that selectively downregulates ICAM-1 and B7–2 and modulates T cell costimulation. J. Clin. Invest.

[95] Sanchez D.J., Gumperz J.E., Ganem D. (2005). Regulation of CD1d expression and function by a herpesvirus infection. J. Clin. Invest.

[96] Mansouri M., Douglas J., Rose P.P., Gouveia K., Thomas G., Means R.E., Moses A.V., Fruh K. (2006). Kaposi sarcoma herpesvirus K5 removes CD31/PECAM from endothelial cells. Blood.

[97] Bartee E., McCormack A., Fruh K. (2006). Quantitative membrane proteomics reveals new cellular targets of viral immune modulators. PLoS Pathog.

[98] Li Q., Means R., Lang S., Jung J.U. (2007). Downregulation of gamma interferon receptor 1 by Kaposi’s sarcoma-associated herpesvirus K3 and K5. J. Virol.

[99] Mansouri M., Rose P.P., Moses A.V., Fruh K. (2008). Remodeling of endothelial adherens junctions by Kaposi’s sarcoma-associated herpesvirus. J. Virol.

[100] Del Pozo J.C., Estelle M. (1999). The Arabidopsis cullin AtCUL1 is modified by the ubiquitin-related protein RUB1. Proc. Natl. Acad. Sci. USA.

[101] Hotton S.K., Callis J. (2008). Regulation of cullin RING ligases. Ann. Rev. Plant Biol.

[102] Lozano-Duran R., Rosas-Diaz T., Gusmaroli G., Luna A.P., Taconnat L., Deng X.W., Bejarano E.R. (2011). Geminiviruses subvert ubiquitination by altering CSN-mediated derubylation of SCF E3 ligase complexes and inhibit jasmonate signaling in Arabidopsis thaliana. Plant Cell.

[103] Greenberg J.T., Yao N. (2004). The role and regulation of programmed cell death in plant-pathogen interactions. Cell Microbiol.

[104] Mo M., Fleming S.B., Mercer A.A. (2009). Cell cycle deregulation by a poxvirus partial mimic of anaphase-promoting complex subunit 11. Proc. Natl. Acad. Sci. USA.

[105] Rodier F., Campisi J., Bhaumik D. (2007). Two faces of p53: Aging and tumor suppression. Nucleic Acids Res.

[106] Momand J., Wu H.H., Dasgupta G. (2000). MDM2—master regulator of the p53 tumor suppressor protein. Gene.

[107] Fang S., Jensen J.P., Ludwig R.L., Vousden K.H., Weissman A.M. (2000). Mdm2 is a RING finger-dependent ubiquitin protein ligase for itself and p53. J. Biol. Chem.

[108] Honda R., Yasuda H. (2000). Activity of MDM2, a ubiquitin ligase, toward p53 or itself is dependent on the RING finger domain of the ligase. Oncogene.

[109] Manning A.L., Dyson N.J. (2011). pRB, a tumor suppressor with a stabilizing presence. Trends Cell Biol.

[110] Chellappan S., Kraus V.B., Kroger B., Munger K., Howley P.M., Phelps W.C., Nevins J.R. (1992). Adenovirus E1A, simian virus 40 tumor antigen, and human papillomavirus E7 protein share the capacity to disrupt the interaction between transcription factor E2F and the retinoblastoma gene product. Proc. Natl. Acad. Sci. USA.

[111] Demeret C., Desaintes C., Yaniv M., Thierry F. (1997). Different mechanisms contribute to the E2-mediated transcriptional repression of human papillomavirus type 18 viral oncogenes. J. Virol.

[112] Thierry F., Yaniv M. (1987). The BPV1-E2 trans-acting protein can be either an activator or a repressor of the HPV18 regulatory region. EMBO J.

[113] Bellanger S., Tan C.L., Nei W., He P.P., Thierry F. (2010). The human papillomavirus type 18 E2 protein is a cell cycle-dependent target of the SCFSkp2 ubiquitin ligase. J. Virol.

[114] Blachon S., Bellanger S., Demeret C., Thierry F. (2005). Nucleo-cytoplasmic shuttling of high risk human Papillomavirus E2 proteins induces apoptosis. J. Biol. Chem.

[115] Gagnon D., Joubert S., Senechal H., Fradet-Turcotte A., Torre S., Archambault J. (2009). Proteasomal degradation of the papillomavirus E2 protein is inhibited by overexpression of bromodomain-containing protein 4. J. Virol.

[116] Penrose K.J., McBride A.A. (2000). Proteasome-mediated degradation of the papillomavirus E2-TA protein is regulated by phosphorylation and can modulate viral genome copy number. J. Virol.

[117] Han Y.H., Xiang H.Y., Wang Q., Li Y.Y., Wu W.Q., Han C.G., Li D.W., Yu J.L. (2010). Ring structure amino acids affect the suppressor activity of melon aphid-borne yellows virus P0 protein. Virology.

[118] Pfeffer S., Dunoyer P., Heim F., Richards K.E., Jonard G., Ziegler-Graff V. (2002). P0 of *Beet western yellows virus* is a suppressor of posttranscriptional gene silencing. J. Virol.

[119] Pazhouhandeh M., Dieterle M., Marrocco K., Lechner E., Berry B., Brault V., Hemmer O., Kretsch T., Richards K.E., Genschik P. (2006). F-box-like domain in the polerovirus protein P0 is required for silencing suppressor function. Proc. Natl. Acad. Sci. USA.

[120] Bernstein E., Denli A.M., Hannon G.J. (2001). The rest is silence. RNA.

[121] Hamilton A.J., Baulcombe D.C. (1999). A species of small antisense RNA in posttranscriptional gene silencing in plants. Science.

[122] Hammond S.M., Bernstein E., Beach D., Hannon G.J. (2000). An RNA-directed nuclease mediates post-transcriptional gene silencing in *Drosophila* cells. Nature.

[123] Van Wolfswinkel J.C., Ketting R.F. (2010). The role of small non-coding RNAs in genome stability and chromatin organization. J. Cell Sci.

[124] Baumberger N., Baulcombe D.C. (2005). Arabidopsis ARGONAUTE1 is an RNA Slicer that selectively recruits microRNAs and short interfering RNAs. Proc. Natl. Acad. Sci. USA.

[125] Akbergenov R., Si-Ammour A., Blevins T., Amin I., Kutter C., Vanderschuren H., Zhang P., Gruissem W., Meins F., Hohn T. (2006). Molecular characterization of geminivirus-derived small RNAs in different plant species. Nucleic Acids Res.

[126] Bouche N., Lauressergues D., Gasciolli V., Vaucheret H. (2006). An antagonistic function for Arabidopsis DCL2 in development and a new function for DCL4 in generating viral siRNAs. EMBO J.

[127] Deleris A., Gallego-Bartolome J., Bao J., Kasschau K.D., Carrington J.C., Voinnet O. (2006). Hierarchical action and inhibition of plant Dicer-like proteins in antiviral defense. Science.

[128] Moissiard G., Voinnet O. (2006). RNA silencing of host transcripts by cauliflower mosaic virus requires coordinated action of the four Arabidopsis Dicer-like proteins. Proc. Natl. Acad. Sci. USA.

[129] Zhang X.R., Yuan Y.R., Pei Y., Lin S.S., Tuschl T., Patel D.J., Chua N.H. (2006). Cucumber mosaic virus-encoded 2b suppressor inhibits Arabidopsis Argonaute1 cleavage activity to counter plant defense. Genes Dev.

[130] Shimura H., Pantaleo V. (2011). Viral induction and suppression of RNA silencing in plants. Biochim. Biophys. Acta.

[131] Mlotshwa S., Pruss G.J., Peragine A., Endres M.W., Li J., Chen X., Poethig R.S., Bowman L.H., Vance V. (2008). DICER-LIKE2 plays a primary role in transitive silencing of transgenes in Arabidopsis. PLoS One.

[132] Burgyan J., Havelda Z. (2011). Viral suppressors of RNA silencing. Trends Plant Sci.

[133] Van der Wilk F., Houterman P., Molthoff J., Hans F., Dekker B., van den Heuvel J.F.J.M., Huttinga H., Goldbach R. (1997). Expression of the potato leafroll virus ORFO induces viral-disease-like symptoms in transgenic potato plants. Mol. Plant Microbe Interact.

[134] Kozlowska-Makulska A., Guilley H., Szyndel M.S., Beuve M., Lemaire O., Herrbach E., Bouzoubaa S. (2010). P0 proteins of European beet-infecting poleroviruses display variable RNA silencing suppression activity. J. Gen. Virol.

[135] Mangwende T., Wang M.L., Borth W., Hu J., Moore P.H., Mirkov T.E., Albert H.H. (2009). The P0 gene of Sugarcane yellow leaf virus encodes an RNA silencing suppressor with unique activities. Virology.

[136] Baumberger N., Tsai C.H., Lie M., Havecker E., Baulcombe D.C. (2007). The polerovirus silencing suppressor P0 targets Argonaute proteins for degradation. Curr. Biol.

[137] Bortolamiol D., Pazhouhandeh M., Marrocco K., Genschik P., Ziegler-Graff V. (2007). The polerovirus F box protein P0 targets Argonaute1 to suppress RNA silencing. Curr. Biol.

[138] Csorba T., Lozsa R., Hutvagner G., Burgyan J (2010). Polerovirus protein P0 prevents the assembly of small RNA containing RISC complexes and leads to degradation of ARGONAUTE1. Plant J..

[139] Berry B., Deddouche S., Kirschner D., Imler J.L., Antoniewski C. (2009). Viral suppressors of RNA silencing hinder exogenous and endogenous small RNA pathways in Drosophila. PLoS One.

[140] Ye C., Verchot J. (2011). Role of unfolded protein response in plant virus infection. Plant Signaling Behav.

